# Peritumor to tumor apparent diffusion coefficient ratio is associated with biologically more aggressive breast cancer features and correlates with the prognostication tools

**DOI:** 10.1371/journal.pone.0235278

**Published:** 2020-06-25

**Authors:** Hidemi Okuma, Mazen Sudah, Tiia Kettunen, Anton Niukkanen, Anna Sutela, Amro Masarwah, Veli-Matti Kosma, Päivi Auvinen, Arto Mannermaa, Ritva Vanninen

**Affiliations:** 1 Institute of Clinical Medicine, School of Medicine, Clinical Radiology, University of Eastern Finland, Kuopio, Finland; 2 Department of Clinical Radiology, Diagnostic Imaging Center, Kuopio University Hospital, Kuopio, Finland; 3 Institute of Clinical Medicine, School of Medicine, Oncology, University of Eastern Finland, Kuopio, Finland; 4 Department of Oncology, Cancer Center, Kuopio University Hospital, Kuopio, Finland; 5 Institute of Clinical Medicine, School of Medicine, Pathology and Forensic Medicine, and Translational Cancer Research Area, University of Eastern Finland, Kuopio, Finland; 6 Biobank of Eastern Finland, Kuopio University Hospital, Kuopio, Finland; Medical University of Vienna, AUSTRIA

## Abstract

**Purpose:**

The apparent diffusion coefficient (ADC) is increasingly used to characterize breast cancer. The peritumor/tumor ADC ratio is suggested to be a reliable and generally applicable index. However, its overall prognostication value remains unclear. We aimed to evaluate the associations between the peritumor/tumor ADC ratio and histopathological biomarkers and published prognostic tools in patients with invasive breast cancer.

**Materials and methods:**

This prospective study included 88 lesions (five bilateral) in 83 patients with primary invasive breast cancer who underwent preoperative 3.0-T magnetic resonance imaging. The lowest intratumoral mean ADC value on the slice with the largest tumor cross-sectional area was designated the tumor ADC, and the highest mean ADC value on the peritumoral breast parenchymal tissue adjacent to the tumor border was designated the peritumor ADC. The peritumor/tumor ADC ratio was then calculated. The tumor and peritumor ADC values and peritumor/tumor ADC ratios were compared with histopathological parameters using an unpaired *t* test, and their correlations with published prognostic tools were evaluated with Pearson’s correlation coefficient.

**Results:**

The peritumor/tumor ADC ratio was significantly associated with tumor size (p<0.001), histological grade (p = 0.005), Ki-67 index (p = 0.006), axillary-lymph-node metastasis (p = 0.001), and lymphovascular invasion (p = 0.006), but was not associated with estrogen receptor status (p = 0.931), progesterone receptor status (p = 0.160), or human epidermal growth factor receptor 2 status (p = 0.259). The peritumor/tumor ADC ratio showed moderate positive correlations with the Nottingham Prognostic Index (*r* = 0.498, p<0.001) and mortality predicted using PREDICT (*r* = 0.436, p<0.001).

**Conclusion:**

The peritumor/tumor ADC ratio was correlated with histopathological biomarkers in patients with invasive breast cancer, showed significant correlations with published prognostic indexes, and may provide an easily applicable imaging index for the preoperative prognostic evaluation of breast cancer.

## Introduction

The peritumoral area, which immediately surrounds the tumor, consists of extracellular matrix with various cell types including fibroblasts, endothelial cells, and infiltrating leukocytes [1]. Although the details of the biologic mechanisms underlying the presence of peritumoral edema remains unclear, it is suggested that proteolysis and neoangiogenesis associated with invasive growth and tumor progression triggers the release of cytokines and an increase of vascular permeability, which consequently induces transudation of fluid in the peritumoral area [2]. Several studies have shown that peritumoral edema is associated with tumor aggressiveness and worse prognosis [3–5]. Also, gene signature in the peritumoral stroma has been shown to be distinct from that of the tumoral stroma [6–8]. Thus the peritumoral area represents a unique microenvironment that has independent prognostic potential from intratumoral area [9].

Diffusion-weighted imaging (DWI) is a key noninvasive functional imaging technique, which exploits the random motion of water molecules, and is sensitive to tissue microstructure and cellularity. The apparent diffusion coefficient (ADC) is a quantitative measure of diffusion that is increasingly used to characterize and discriminate lesions [10]. Although some challenges to improve the generalizability and reproducibility of breast DWI [11], the limited reproducibility of ADC across different imaging manufacturers, field strengths, and imaging centers is contentious [12, 13]. To reduce the effects of possible equipment-related confounding factors, the peritumor/tumor ADC ratio may be a more reliable and generally more applicable tool than just the tumoral or peritumoral areas. However, few studies have examined the associations between the peritumor/tumor ADC ratio and the biological and histological features of breast cancers [4, 14]. Furthermore, the overall prognostication value of the peritumor/tumor ADC ratio remains unclear.

Because aggressive breast cancers have higher peritumor ADC values, reflecting the peritumoral edema, and lower tumor ADC values, reflecting the increased cell density caused by proliferative changes, we hypothesized that a greater peritumor/tumor ADC ratio in invasive breast cancer would better correlate with traditional histopathological prognostic factors than either of the individual measures alone. The peritumor/tumor ADC ratio should also correlate with other published prognostic models.

The Nottingham Prognostic Index (NPI), which combines nodal status, tumor size, and histological grade in a simple formula, was first introduced in 1982 [15]. Its advantage in prognostic discrimination has been validated in several large studies [16, 17] and it has been a widely accepted clinical tool for calculating an individual’s prognosis for decades. PREDICT [18] is a freely available web-based prognostic tool created for breast cancer prognostication and to predict treatment benefits according to clinical and histopathological parameters. Although its validation has been extensively discussed [19, 20], PREDICT is increasingly used by clinicians and plays an important role in individualized prognoses in the practice of precision medicine [21].

The purpose of this study was to evaluate whether the peritumor/tumor ADC ratio is associated with traditional histopathological biomarkers and published prognostic indexes, such as NPI and the predicted mortality calculated with PREDICT, in patients with invasive breast cancer.

## Materials and methods

**Fig 1:**
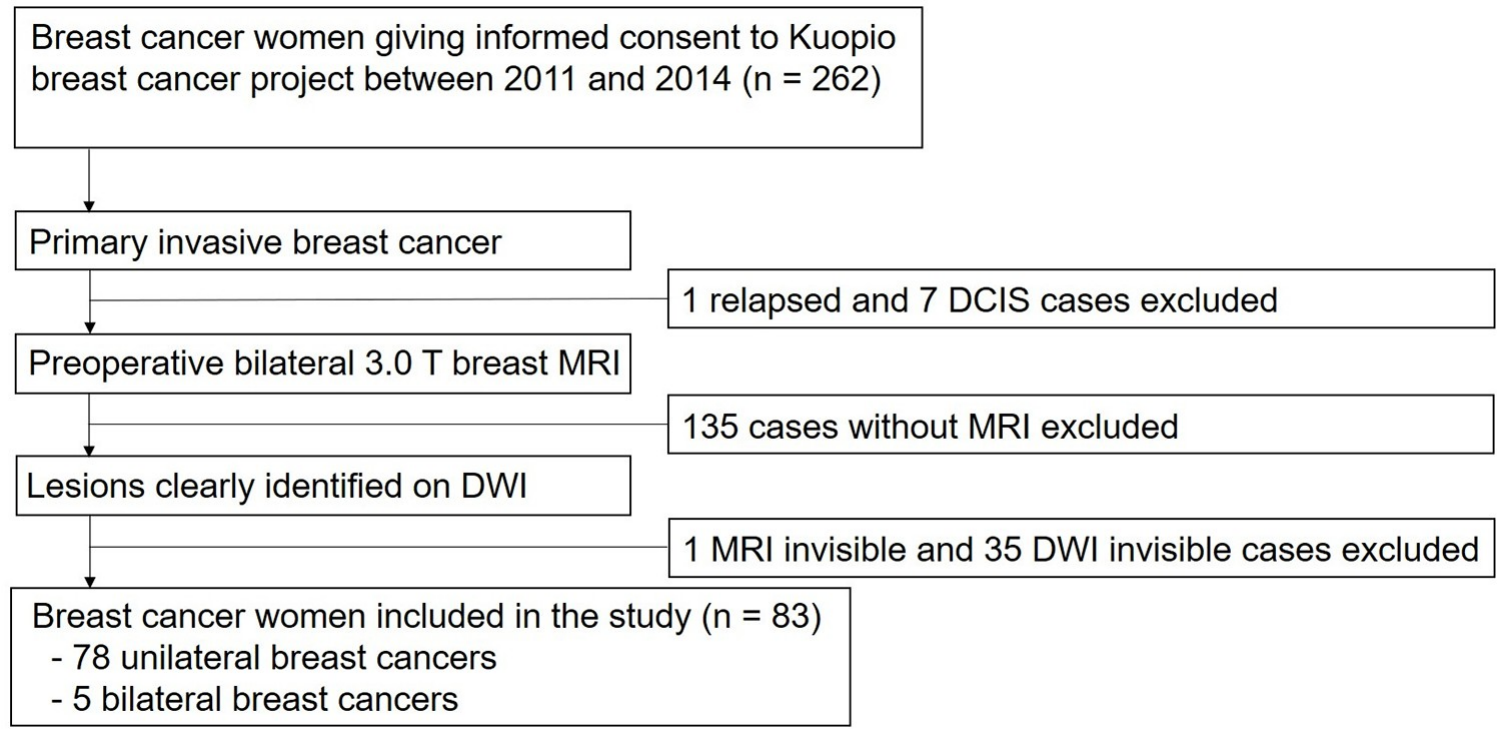
Flowchart of the study population. DCIS = ductal carcinoma in situ, DWI = diffusion-weighted imaging.

This study was based on a database of 262 consecutive breast cancer patients prospectively included in a translational breast cancer study in 2011–2014 at Kuopio University Hospital, where patients from local screening centers, district hospitals, and tertiary care centers are referred to for management of clinically or screening-detected breast lesions. Of these patients, the current study included women with breast cancer who 1) were newly diagnosed with invasive breast cancer; 2) underwent preoperative bilateral 3.0-T breast magnetic resonance imaging (MRI); and 3) had lesions clearly demarcated on diffusion-weighted imaging (DWI). At our institution, breast MRI is not performed routinely in all patients, but performed in accordance with the guidelines of the European Society of Breast Cancer Specialists (EUSOMA) working group [22]. Briefly, the indications for breast MRI include staging in problematic situations before final treatment planning, characterization of equivocal findings at conventional imaging and invasive lobular cancer. A total of 88 lesions (five bilateral) in 83 patients were included as the study cohort (Fig 1). Adjuvant treatments were given according to the national guidelines, which are in accordance with the international guidelines [23, 24]. Written informed consent was obtained from all the patients before any procedures were performed and all data were fully anonymized before accessing. The study was approved by the Research Ethics Board of Kuopio University Hospital and all clinical investigations were conducted according to the relevant guidelines and the principles expressed in the Declaration of Helsinki.

### MRI

MRI examinations were performed in the prone position with a seven-element phased-array coil dedicated to breast imaging (Philips Achieva 3.0 T TX, Philips N.V., Eindhoven, The Netherlands). The structural breast MRI protocol consisted of five sequences (Table 1). DWI was performed with five respective b factors (0, 200, 400, 600, and 800 s/mm^2^). ADC maps were automatically calculated linearly with the method provided by the MRI manufacturer.

**Table 1:** Breast MRI protocol.

Sequence	TR/TE (ms)	In-plane Resolution (mm)	Slice Thickness (mm)	Scanning Time (seconds)
T1-FFE	4.58 / 2.3	0.48 × 0.48	0.7	371
T2-TSE	5000 / 120	0.6 × 0.6	2	200
STIR	5000 / 60	1 × 1	2	340
T1 dynamic *	4.67 / 2.31	0.96 × 0.96	1	58.5
DWI #	7168 / 95	1.15 × 1.15	4	151

* eTHRIVE spectrally adiabatic inversion recovery (SPAIR) fat suppression; precontrast and six phases after the gadoterate meglumine injection (0.2 ml/kg, 3 ml/s) followed by a saline chaser

^#^ DWI: diffusion-weighted echo planar imaging with five respective b factors (0, 200, 400, 600, and 800 s/mm^2^)

FFE = fast field echo, TSE = turbo spin echo, STIR = short tau inversion recovery, TR = repetition time, TE = echo time

### Image interpretation

T1-weighted, T2-weighted, and dynamic contrast-enhanced images were used as references, and a crosshair tool (Sectra PACS, version 15.1.20.2, Sectra Workstation IDS7, Linköping, Sweden) was used to locate the tumor and correctly position the region of interest (ROI) on the ADC map. The largest tumor cross-sectional area was selected on the ADC map, and three round ROIs (4 pixels per ROI) were placed inside the tumor where the ADC values appeared (visually) to be most strongly reduced. This protocol was based on a recent study that demonstrated that smaller tumor ROIs in the subregions with the most restricted diffusion more accurately show the aggressiveness of the tumor and better correlate with prognostic factors than the entire tumor ROI [25]. Cystic, necrotic, fatty, and hemorrhagic areas were carefully avoided. The mean ADC values were recorded from each ROI, and the lowest value among the three was designated the tumor ADC.

**Fig 2:**
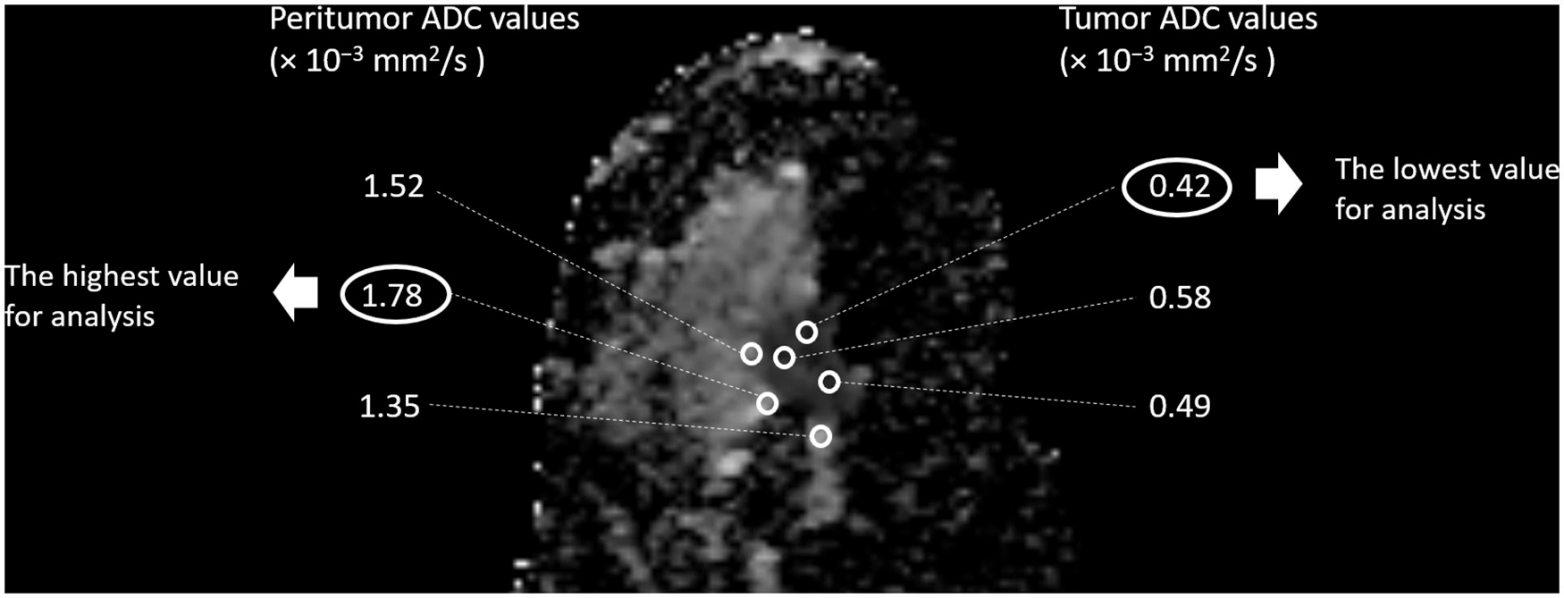
Methods used to measure the tumor and peritumor apparent diffusion coefficient (ADC) values. The ADC map on the slice with the largest tumor cross-sectional area was selected for measurement, and three round regions of interest (ROIs) were placed inside the tumor where the ADC values appeared (visually) to be most strongly reduced. Then, three round ROIs were placed on the peritumoral breast parenchymal tissue adjacent to the tumor border at locations at which the ADC values appeared (visually) to be most strongly increased. The lowest of the three mean tumor ADC values (0.43 × 10^−3^ mm^2^/s) and the highest of the three mean peritumor ADC values (1.78 × 10^−3^ mm^2^/s) were selected for further analysis and for the calculation of the peritumor/tumor ADC ratio ([1.78 × 10^−3^]/[0.43 × 10^−3^] = 4.18).

**Fig 3:**
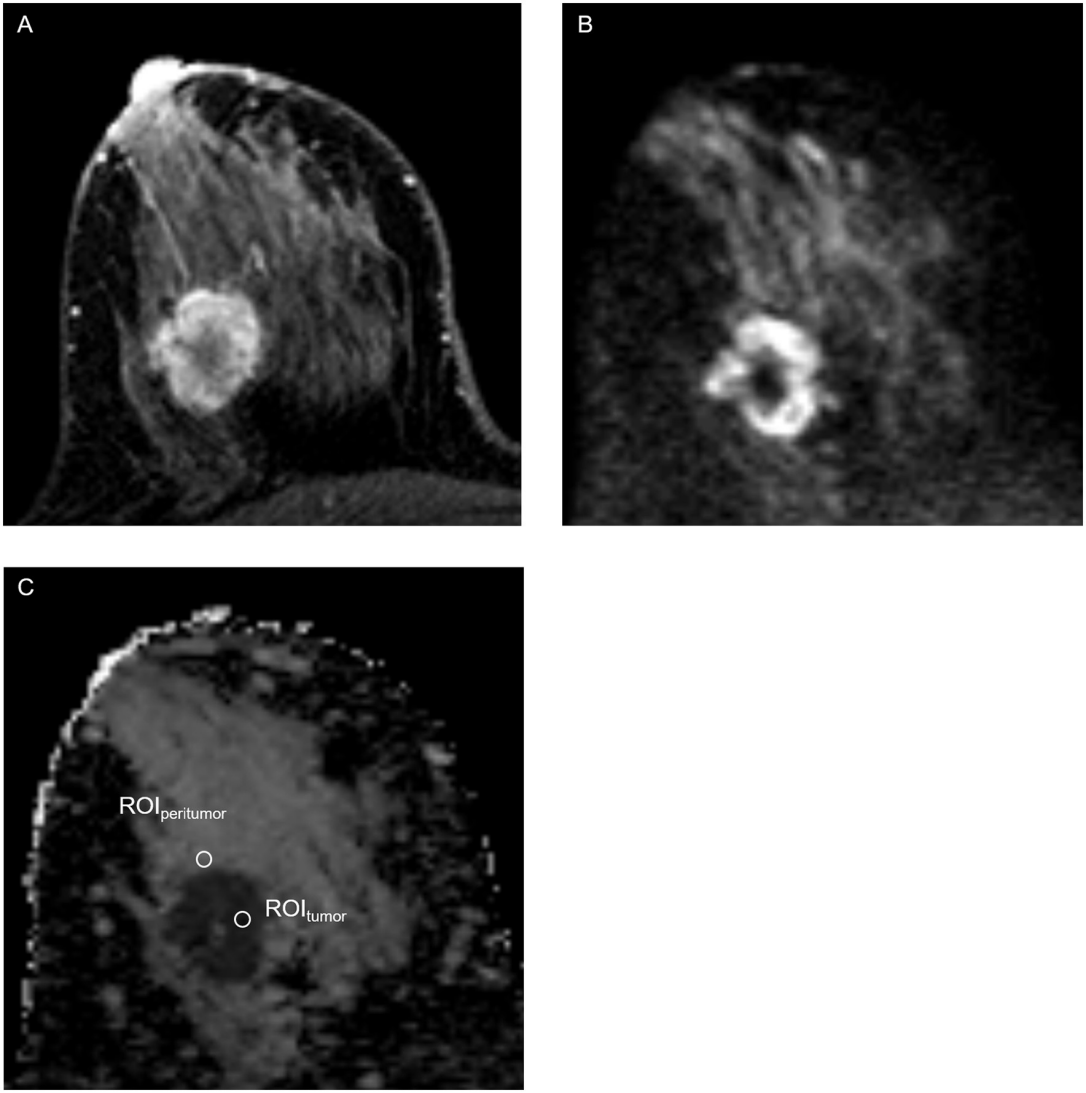
Example of a breast lesion in a 41-year-old female patient with a high peritumor/tumor apparent diffusion coefficient (ADC) ratio. A, Axial T1-weighted gadolinium-enhanced thin slice source image shows a mass with rim enhancement in the right breast; B, High intensity signal is shown in axial diffusion-weighted image (*b* value = 800 s/mm^2^); C, ADC map shows restricted diffusion in the mass. The lowest mean tumor ADC value (shown as ROI_tumor_) was 0.42 × 10^−3^ mm^2^/s and the highest mean peritumor ADC value (shown as ROI_peritumor_) was 2.05 × 10^−3^ mm^2^/s. The peritumor/tumor ADC ratio was 4.86. The clinical and histopathological features of the lesion were: pT2N1, grade 3, human epidermal growth factor receptor 2 negative, estrogen receptor and progesterone receptor positive, Ki-67 high (≥20%), lymphovascular invasion negative. The patient underwent breast conserving surgery and axillary lymph node dissection.

**Fig 4:**
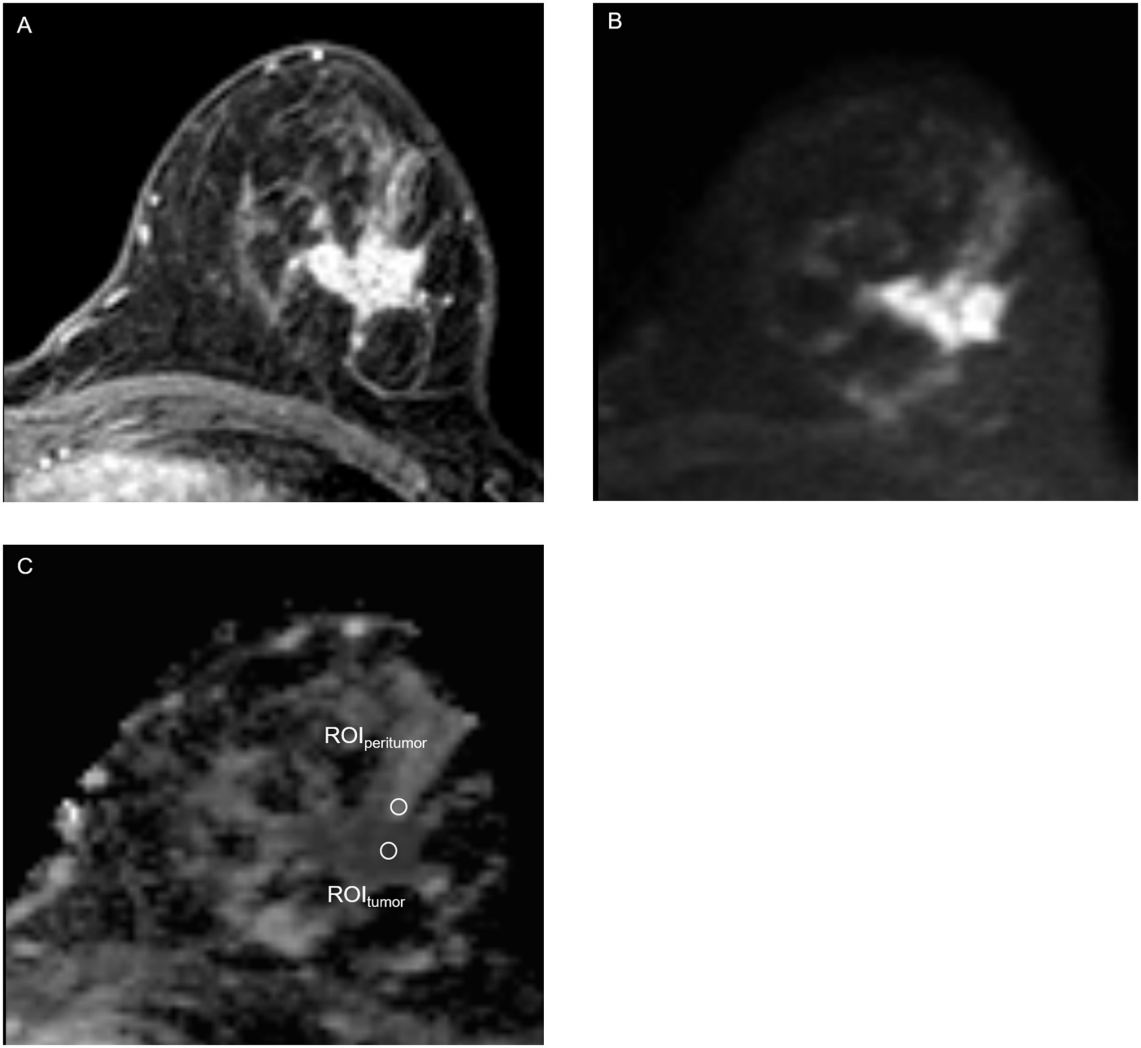
Example of a breast lesion in a 44-year-old female patient with a low peritumor/tumor apparent diffusion coefficient (ADC) ratio. A, Axial T1-weighted gadolinium-enhanced thin slice source image shows an irregular heterogeneously enhancing mass in the left breast; B, High intensity signal is shown in axial diffusion-weighted image (*b* value = 800 s/mm^2^); C, ADC map shows restricted diffusion in the mass. The lowest mean tumor ADC value (shown as ROI_tumor_) was 0.69 × 10^−3^ mm^2^/s and the highest mean peritumor ADC value (shown as ROI_peritumor_) was 1.32 × 10^−3^ mm^2^/s. The peritumor/tumor ADC ratio was 1.91. The clinical and histopathological features of the lesion were: pT2N0sn, grade 3, human epidermal growth factor receptor 2 positive, estrogen receptor and progesterone receptor positive, Ki-67 high (≥20%), lymphovascular invasion positive. The patient underwent mastectomy and sentinel lymph node biopsy.

Three round ROIs (4 pixels per ROI) were then placed on the peritumoral breast parenchymal tissue adjacent to the tumor border at locations at which the ADC values appeared (visually) to be most strongly increased [4, 14]. The mean ADC values were recorded for each ROI, and the highest value among the three was designated the peritumor ADC. The peritumor/tumor ADC ratio was then calculated. A schematic illustration of the tumor and peritumor ROIs is shown in Fig 2. Illustrative images of tumors with high and low peritumor/tumor ADC ratios are shown in Figs 3 and 4, respectively.

A breast radiologist (HO, with 10 years of experience in breast MRI) and a breast oncologist (TK, with 4 years of experience in breast MRI) measured the tumor and peritumor ADC values while blinded to all clinical and pathological information. The breast radiologist evaluated the ADC values twice, with an 8-month interval between the measurements.

### Histopathological assessment

Histopathological samples were obtained from preoperative core needle biopsy (CNB) and surgical specimens, and were evaluated by senior pathologists according to the World Health Organization Classification of Tumors of the Breast [26]. An immunohistochemical analysis of the tumor was performed to detect the estrogen receptor (ER), progesterone receptor (PR), and human epidermal growth factor receptor 2 (HER2). Lymphovascular invasion (LVI) was also assessed on hematoxylin-and-eosin-stained sections. Micrometastases were counted as node positive, while isolated tumor cells were counted as node negative. The histopathological data, including the tumor size, histological grade, ER status, PR status, HER2 status, Ki-67 index, and the presence of axillary-lymph-node metastasis (LNM) and LVI, were obtained from histopathological reports. The tumor size was dichotomized to large (≥ T2) or small (≤ T1c). The tumor grade was dichotomized as high (grade 3) or low (grade 1 or 2). Ki-67 expression was dichotomized as high (≥ 20%) or low (< 20%).

### Prognostic tools

NPI was calculated with the formula: NPI = tumor size (cm) × 0.2 + histological grade (1–3) + lymph-node scores (negative node = 1; 1–3 positive nodes = 2; ≥ 4 positive nodes = 3) [27]. The predicted overall 10-year mortality was calculated for each patient individually by entering the prognosticators into the web interface PREDICT version 2.1 (http://www.predict.nhs.uk). The data entered into the program included age at diagnosis, tumor size, tumor grade, number of positive nodes, ER status, HER2 status, Ki-67 status, mode of detection of breast cancer, and chemotherapy regimen used for adjuvant treatment.

### Statistical analysis

All statistical analyses were performed with SPSS version 22 (IBM Corporation, Armonk, NY, USA). The ADC values were evaluated as continuous dependent variables and expressed as means ± standard deviations.

Intra- and interobserver reproducibility was evaluated using the intraclass correlation coefficients (ICCs). An *r* of 1.0 was considered as perfect agreement; 0.81–0.99 as almost perfect; 0.61–0.80 as substantial; 0.41–0.60 as moderate; 0.21–0.40 as fair, and ≦ 0.20 as slight agreement [28].

An unpaired *t* test was used to compare the tumor ADC values, peritumor ADC values, and peritumor/tumor ADC ratios with the dichotomized histopathological parameters (tumor size, histological grade, ER, PR, HER2, Ki-67, LNM, and LVI). Diagnostic performance of the peritumor/tumor ADC ratios in prediction of final histopathological parameters of breast cancers was separately calculated for each dichotomized histopathological parameter. The optimal threshold values were first selected using Receiver Operating Characteristics analysis. Sensitivity, specificity and positive likelihood ratios (LR+) were then calculated for those histopathological parameters with statistical significance in univariate analysis. The Kaplan-Meier method with log rank (Mantel-Cox) test was used to compare survival in dichotomized groups of tumor ADC values (≥ or < 0.50 × 10^−3^ mm^2^/s), peritumor ADC values (≥ or < 1.75 × 10^−3^ mm^2^/s) and peritumor/tumor ADC ratios (≥ or < 3.5). The correlations between tumor ADC values, peritumor ADC values, and peritumor/tumor ADC ratios and published prognostic indexes (NPI and predicted mortality calculated with PREDICT), as well as cross correlation among continuous histopathological parameters were evaluated with Pearson’s correlation coefficient. P values ≤ 0.05 were considered to be statistically significant. Correlation coefficients of *r* ≤ 0.35 were considered to be weak, 0.36–0.67 as moderate, and ≥ 0.68 as strong [29].

## Results

Eighty-three women (mean age 57.5 ± 11.2 years, range 28–81 years) with 88 invasive (five bilateral) breast cancers were analyzed. Their patient profiles and tumor characteristics are described in Table 2.

**Table 2:** Patient profiles and tumor characteristics.

Characteristic	N (%)
**Patients / Lesions**	83 / 88
**Age (years)**	57.5 ± 11.2
**Menopause status**	
premenopause	31 (37.3)
postmenopause	52 (62.7)
**Tumor stage**	
pT1	50 (56.8)
pT2	35 (39.8)
pT3	3 (3.4)
pT4	0 (0)
**Axillary node classification**	
pN0	49 (55.7)
pN1	23 (26.1)
pN2	13 (14.8)
pN3	3 (3.4)
**Histological grade**	
G1	12 (13.6)
G2	49 (55.7)
G3	27 (30.7)
**Human epidermal growth factor receptor 2**	
positive	19 (21.6)
negative	69 (78.4)
**Estrogen receptor**	
positive	82 (93.2)
negative	6 (6.8)
**Progesterone receptor**	
positive	79 (89.8)
negative	9 (10.2)
**Ki-67 expression**	
< 20%	47 (53.4)
≥ 20%	41 (46.6)
**Tumor type**	
ductal (no special type)	67 (76.1)
lobular	18 (20.5)
others	3 (3.4)
**Prognostic scores**	
Nottingham Prognostic Index	2.0–7.8 (median 4.0)
Mortality predicted with PREDICT	6–86 (median 20)
**Surgery**	
total mastectomy	36 (40.9)
breast conserving surgery	52 (59.1)
**Adjuvant treatment**	
chemotherapy	59 (71.1)
hormonal treatment	65 (78.3)
trastuzumab	17 (20.4)
postoperative radiotherapy	68 (82.0)

In the evaluation of the agreement within and across readers, ICCs for tumoral (r = 0.925 and 0.910, respectively) and peritumoral (r = 0.951 and 0.945, respectively) ADC measurements all exceeded 0.81, indicating almost perfect agreement (Table 3).

**Table 3:** Interclass Correlation Coefficients (ICCs) of ADC.

	Intra-observer ICCs (95% confidence interval)	Inter-observer ICCs (95% confidence interval)
Tumoral ADC	0.925 (0.886–0.951)	0.910 (0.863–0.941)
Peritumoral ADC	0.951 (0.925–0.968)	0.945 (0.916–0.964)

The tumor ADC value was significantly associated with tumor size (p = 0.021) and the Ki-67 index (p = 0.035), but was not associated with the histological grade (p = 0.103), ER status (p = 0.296), PR status (p = 0.324), HER2 status (p = 0.381), axillary LNM (p = 0.111), or LVI (p = 0.769).

The peritumor ADC value was significantly associated with axillary LNM (p = 0.012) and LVI (p = 0.010), but was not associated with tumor size (p = 0.058), histological grade (p = 0.123), ER status (p = 0.105), PR status (p = 0.525), HER2 status (p = 0.409), or the Ki-67 index (p = 0.513).

The peritumor/tumor ADC ratio was significantly associated with tumor size (p < 0.001), histological grade (p = 0.005), Ki-67 index (p = 0.006), axillary LNM (p = 0.001), and LVI (p = 0.006), but was not associated with ER status (p = 0.931), PR status (p = 0.160), or HER2 status (p = 0.259). The associations of the tumor and peritumor ADC values and the peritumor/tumor ADC ratios with the histopathological parameters are presented in Table 4.

**Table 4:** Associations between tumor and peritumor ADC values, peritumor/tumor ADC ratios, and histopathological parameters.

	Tumor		Peritumor		peritumor-tumor ADC ratios	
	ADC values (× 10^−3^ mm^2^ /s)	p value	ADC values (× 10^−3^ mm^2^ /s)	p value	ADC ratios	p value
**Tumor size**						
large (≥ T2)	0.49 ± 0.17	0.021	1.67 ± 0.53	0.058	3.64 ± 1.33	< 0.001
small (≤ T1c)	0.59 ± 0.21		1.47 ± 0.38		2.71 ± 0.98	
**Histological grade**						
High (3)	0.50 ± 0.16	0.103	1.67 ± 0.49	0.123	3.68 ± 1.37	0.005
Low (1–2)	0.57 ± 0.21		1.50 ± 0.44		2.88 ± 1.09	
**ER**						
positive	0.54 ± 0.20	0.296	1.53 ± 0.45	0.105	3.12 ± 1.22	0.931
negative	0.63 ± 0.14		1.87 ± 0.53		3.17 ± 1.53	
**PR**						
positive	0.55 ± 0.20	0.324	1.55 ± 0.46	0.525	3.03 ± 1.13	0.160
negative	0.49 ± 0.21		1.65 ± 0.47		3.95 ± 1.77	
**HER2**						
positive	0.51 ± 0.17	0.381	1.64 ± 0.47	0.409	3.43 ± 1.08	0.259
negative	0.56 ± 0.20		1.54 ± 0.46		3.05 ± 1.26	
**Ki-67**						
High (≥ 20%)	0.51 ± 0.20	0.035	1.58 ± 0.48	0.513	3.42 ± 1.30	0.006
Low (< 20%)	0.60 ± 0.18		1.51 ± 0.44		2.70 ± 0.99	
**LNM**						
positive	0.51 ± 0.18	0.111	1.69 ± 0.48	0.012	3.60 ± 1.29	0.001
negative	0.58 ± 0.21		1.44 ± 0.42		2.72 ± 1.03	
**LVI**						
positive	0.54 ± 0.18	0.769	1.71 ± 0.47	0.010	3.54 ± 1.33	0.006
negative	0.55 ± 0.21		1.46 ± 0.43		2.83 ± 1.08	

ADC = apparent diffusion coefficient, ER = estrogen receptor, PR = progesterone receptor, HER2 = human epidermal growth factor receptor 2, LNM = lymph node metastasis, LVI = lymphovascular invasion

When we analyzed the ability of the peritumor/tumor ADC ratios to predict the final histopathological parameters of breast cancers, the peritumor/tumor ADC ratios best differentiated between tumors with or without axillary lymph node metastasis (LR+ = 4.57), while they played little role in differentiating between high and low Ki-67 expression (LR+ = 1.64). The results are shown in Table 5.

**Table 5:** Diagnostic performance of the peritumor/Tumor ADC ratios in prediction of histopathological parameters: Optimal thresholds for peritumor/Tumor ADC ratios, sensitivity, specificity and positive Likelihood Ratio (LR+).

	Optimal thresholds for ADC ratios	Sensitivity (%)	Specificity (%)	LR+
Tumor size (large or small)	2.79	76.3	66.0	2.24
Histological grade (high or low)	3.11	63.0	68.9	2.03
Ki-67 (high or low)	2.58	73.2	55.3	1.64
Presence of Axillary Lymph Node Metastasis	3.62	47.5	89.6	4.57
Presence of Lymphovascular Invasion	3.42	55.9	79.6	2.74

During the mean follow up period of 7.2 years (range 5.1–8.7 years), a total of 6 deaths occurred, 5 of which were from breast cancer and 1 from other causes. With regards to overall survival (OS), patients with higher peritumor/tumor ADC ratios were found to have a worse prognosis than those with lower peritumor/tumor ADC ratios (p = 0.03), while no significant associations were found between OS and individual tumor or peritumor ADC values. The association with disease-free survival did not reach significance by any of tumor or peritumor ADC values or peritumor/tumor ADC ratios.

**Fig 5:**
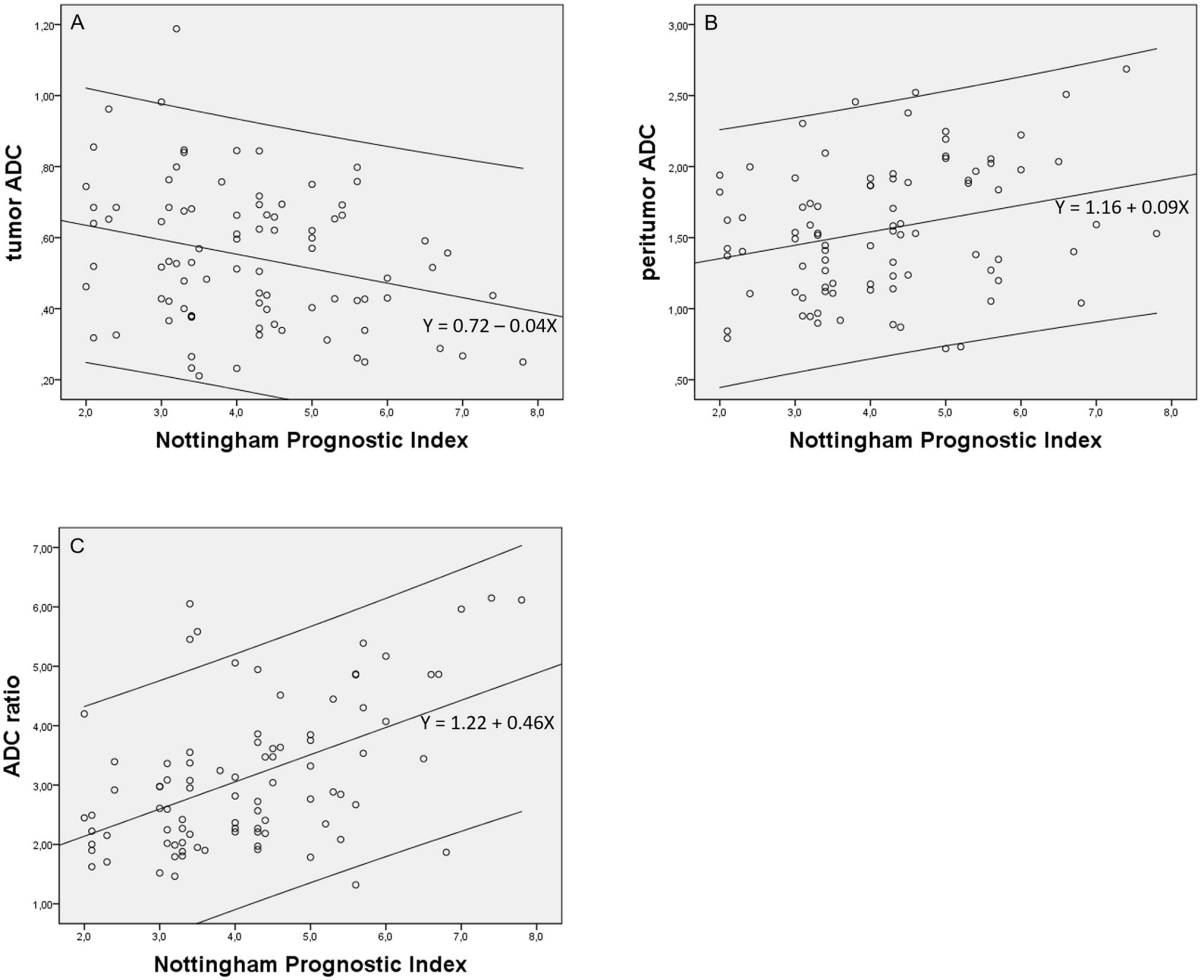
Scatterplots of tumor and peritumor apparent diffusion coefficient (ADC) values and peritumor/tumor ADC ratios against Nottingham Prognostic Index (NPI). Regression lines and 95% confidence intervals are shown. A, Tumor ADC values against NPI (*r* = −0.277, p = 0.009); B, peritumor ADC values against NPI (*r* = 0.273, p = 0.010); C, peritumor/tumor ADC ratios against NPI (*r* = 0.498, p < 0.001).

**Fig 6:**
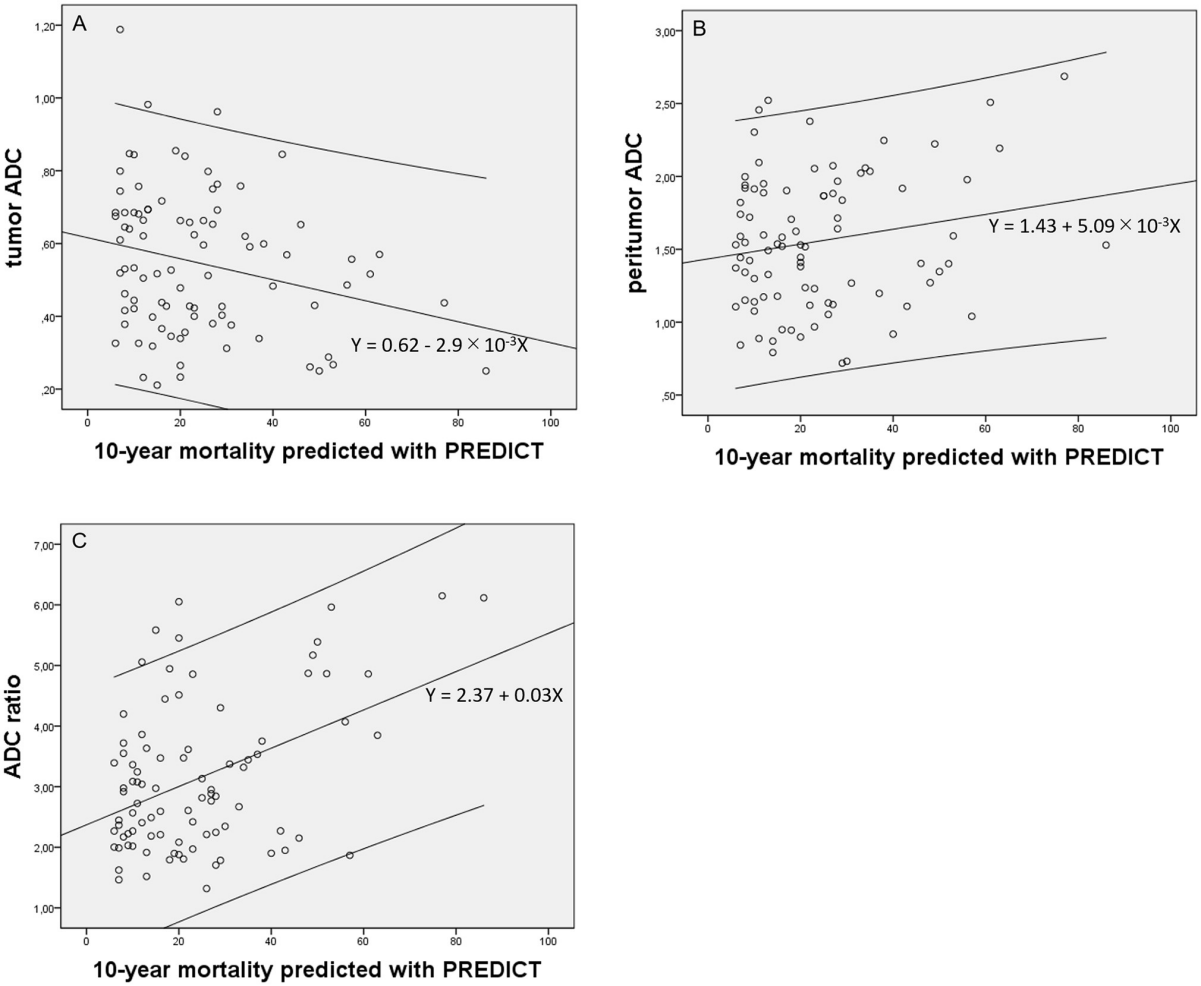
Scatterplots of tumor and peritumor apparent diffusion coefficient (ADC) values and peritumor/tumor ADC ratios against mortality predicted with PREDICT. Regression lines and 95% confidence intervals are shown. A, tumor ADC values against mortality predicted with PREDICT(*r* = −0.250, p = 0.019); B, peritumor ADC values against mortality predicted with PREDICT (*r* = 0.187, p = 0.081); C, peritumor/tumor ADC ratios against mortality predicted with PREDICT (*r* = 0.436, p < 0.001).

The tumor ADC value showed a weak negative correlation with NPI (*r* = −0.277, p = 0.009) and a weak negative correlation with mortality predicted with PREDICT (*r* = −0.250, p = 0.019). The peritumor ADC value showed a weak positive correlation with NPI (*r* = 0.273, p = 0.010), but was not significantly correlated with mortality predicted with PREDICT (*r* = 0.187, p = 0.081). The peritumor/tumor ADC ratio showed moderate positive correlations with both NPI (*r* = 0.498, p < 0.001) and mortality predicted with PREDICT (*r* = 0.436, p < 0.001). NPI and mortality predicted with PREDICT showed a strong positive mutual correlation (*r* = 0.711, p < 0.001). Scatterplots of the tumor and peritumor ADC values and peritumor/tumor ADC ratios against each prognostic tool are shown in Figs 5 and 6.

**Fig 7:**
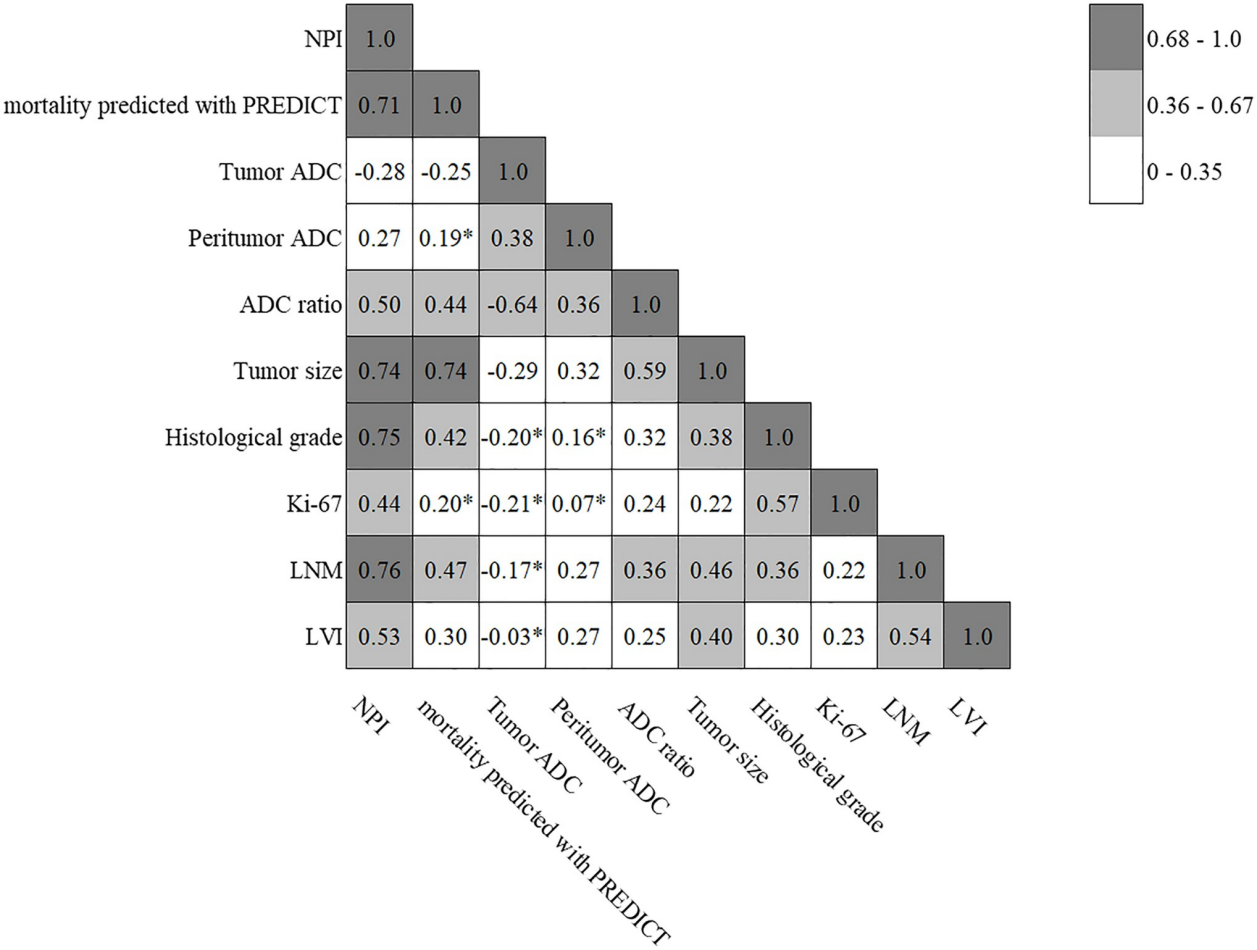
The cross correlation matrix of prognostic tools, tumor and peritumor ADC values, peritumor/tumor ADC ratios and histopathological parameters. Each value represents Pearson’s correlation coefficient. *; statistically non-significant (p > 0.05).

The cross correlation analysis revealed that the peritumor/tumor ADC ratios correlated moderately with tumor size (*r* = 0.59, p < 0.001) and LNM (*r* = 0.36, p = 0.001) yet otherwise weakly with histological grade (*r* = 0.32, p = 0.002), Ki-67 index (*r* = 0.24, p = 0.028) or LVI (*r* = 0.25, p = 0.018), while both tumor and peritumor ADC values correlated only weakly with histopathological parameters. The cross correlation matrix of prognostic tools, tumor and peritumor ADC values, peritumor/tumor ADC ratios and histopathological parameters is presented in Fig 7.

## Discussion

In this study, we separately evaluated the correlations between the ADC values for tumors and peritumoral areas and the peritumor/tumor ADC ratio and the histopathological biomarkers of breast cancer in patients with newly diagnosed invasive breast cancer. A higher peritumor/tumor ADC ratio was associated with a larger tumor diameter, higher tumor grade, higher Ki-67 index, and the presence of axillary LN metastasis and LVI. These associations between traditional prognostic biomarkers and the peritumor/tumor ADC ratio were more significant than the associations of the individual tumor or peritumor ADC values. Paralleling this result, patients with higher peritumor/tumor ADC ratios were found to have a worse prognosis in terms of overall survival, while no significant associations were found between OS and individual tumor or peritumor ADC values. Significant positive correlations were also detected between the peritumor/tumor ADC ratio and published prognostic indexes, such as NPI and PREDICT. Therefore, according to these results, the peritumor/tumor ratio may provide a useful prognostic indicator that is readily available and easily measured on DWI-MRI. Peritumor/tumor ratio performed better than the separate measurements of ADC value from only the tumor or peritumoral area.

DWI helps to differentiate between malignant and benign lesions and thus improves the diagnostic accuracy of MRI. However, previous studies of the prognostic significance of ADC values have generated conflicting outcomes. Discrepancies across studies may be attributable, in part, to the different field strengths or manufacturers of the MRI scanners used [30] or to study designs, which have included different b-values and ROI sizes. Tumor heterogeneity may also influence quantitative imaging parameters when they are analyzed from either the volume of the tumor or the peritumoral area. The molecular effects of the receptor status on tumor angiogenesis and cellularity may also be inconsistent among lesions despite an identical receptor status, as the result of other unmeasured factors [31]. Therefore, to minimize the effects of the factors that confound DWI, we documented the “worst region of interest” by using small ROIs specifically placed where the ADC values appeared to be most reduced inside the tumor and most increased adjacent to the tumor border.

### Associations with histopathological factors

Tumors with larger diameters, higher histological grades, and more metastatic lymph nodes are known to be associated with peritumoral edema [3], which would cause higher peritumor ADC values and result in higher peritumor/tumor ADC ratios. Indeed, in this study, we found that breast cancers with larger diameters, higher histological grades, and LNM were significantly associated with higher peritumor/tumor ADC ratios, whereas peritumor ADC values alone showed lower or no statistically significant associations. Furthermore, the cross correlation analysis revealed that the peritumor/tumor ADC correlated more strongly with larger diameters and presence of LNM among other parameters.

Ki-67 reflects the proliferation rate of various malignant tumors and is an established prognostic or predictive factor in breast cancer [32, 33]. Higher Ki-67 is associated with higher peritumor ADC values [34] and lower tumor ADC values [35], which would together cause a higher peritumor/tumor ADC ratio. This is also consistent with our results. Although Ki-67 was significantly associated with the tumor ADC value (p = 0.035), the peritumor/tumor ratio showed an even more significant association (p = 0.006).

The presence of LVI is another well-established prognostic factor in breast cancer [36] and was previously shown to be associated with the presence of peritumoral edema [37]. LVI was significantly associated with a higher peritumor/tumor ADC ratio in this study, which is consistent with previous results [4, 14].

On the contrary, ER, PR, and HER2 showed no significant correlation with either the tumor or peritumor ADC value or the peritumor/tumor ADC ratio in this study. While some studies found a significant association of tumor ADC value with hormone or HER2 positivity, others found no significant correlations or even inverse relationships [38]. Therefore, the definite effects of hormone and HER2 on tumor ADC values are still controversial. There is only one study investigating the relationship between peritumor ADC value and breast cancer receptor status, where no significant correlation was found between peritumor ADC value and ER or HER2 [39]. Moreover, this is the first study examined the relationship between the peritumor/tumor ADC ratio and breast cancer receptor status. Based on limited evidence, it could be speculated that the HER2 pathway is not necessarily associated with tumor cellularity or edema. Furthermore, because most breast cancers are hormone receptor dependent and the threshold for positivity is set low, we suggest that these relationships should be reevaluated in a larger patient population and with different cut-off values to examine the possibility that there are significant relationships between these parameters in some patient subgroups.

### Correlations with published prognostic indexes

Both NPI and PREDICT are recognized as reliable prognostic tools that facilitate treatment decision-making in the adjuvant setting [40, 41]. Because ADC ratios are associated with factors related to the aggressiveness of breast cancer, such as tumor size and grade, upon which well-established prognostic indexes are based, we investigated the prognostic significance of ADC values and ratios compared with those of well-established prognostic indexes. The peritumor/tumor ADC ratio correlated more strongly with the NPI score and the mortality risk predicted with PREDICT and generally performed better than either the tumor or peritumor ADC value. As expected, NPI and PREDICT showed mutual strong agreement because both methods are based on similar variables. On the contrary, the ADC values measured in either the tumor or the peritumoral area performed poorly. Conflicting results on the prognostic significance of tumor ADC values have previously been attributed to differences in breast anatomy, the locations of the ADC measurements, the devices or protocols used, or mechanical artefacts [42]. However, the better performance of the peritumor/tumor ADC ratio may prove useful in the prognostication of breast cancer or as an additional tool for clinical decision-making.

Although the peritumor/tumor ADC ratios significantly correlated with the established indexes, the correlation was rather limited to moderate. Due to its distinct matrix and gene signature, the peritumoral area can have independent prognostic potential from intratumoral area. Since neither of NPI or PREDICT take the peritumoral factors into account, ADC ratios which incorporate both tumoral and peritumoral factors may have the additional significance in the prognostication which is not covered by the established indexes. Indeed, a recent study showed that peritumoral stiffness is independently prognostic from classical histopathological factors [43].

Longer follow-up with sufficient survival data might show independent significance of ADC ratios on prognostication from classical tumor prognostic factors. Our results suggest that peritumor/tumor ADC ratio is a promising addition to the reporting of multiparametric MRI, because it has the benefit of being available noninvasively before biopsy or operation while it does not require additional time or costs to obtain.

Limitations are that our patient population was relatively small with a limited number of patients with HER2-enriched and triple negative type cancers. The clinical follow-up time is still short, and therefore the outcome measures represent only surrogate markers and survival data should be interpreted with caution. However, in spite of these limitations, the peritumor/tumor ADC ratios proved to have statistically significant prognostic value in terms of overall survival, while the measurements of either tumor or peritumor ADC values alone failed to show any prognostic value. Studies with larger patient samples and longer follow-up period should be conducted to reinforce the conclusions and multivariate analysis should be performed to prove the additional value of the peritumor/tumor ADC ratios.

In summary, the peritumor/tumor apparent diffusion coefficient ratio correlated significantly with histopathological biomarkers such as tumor size, tumor grade, Ki-67 index, presence of lymph-node metastasis, and lymphovascular invasion, as well as with published prognostic indexes, Nottingham Prognostic Index and PREDICT, in patients with invasive breast cancer. These findings suggest that the peritumor/tumor apparent diffusion coefficient ratio is a readily available, easily acquired and applicable imaging index, which works better as a prognostication tool of breast cancer than either the individual tumor or peritumor ADC values.
